# Effects on Parental Stress of Early Home-Based CareToy Intervention in Low-Risk Preterm Infants

**DOI:** 10.1155/2019/7517351

**Published:** 2019-01-22

**Authors:** Giuseppina Sgandurra, Elena Beani, Emanuela Inguaggiato, Jakob Lorentzen, Jens Bo Nielsen, Giovanni Cioni

**Affiliations:** ^1^Department of Developmental Neuroscience, IRCCS Fondazione Stella Maris, Calambrone, Pisa, Italy; ^2^Department of Clinical and Experimental Medicine, University of Pisa, Pisa, Italy; ^3^Department of Neuroscience, University of Copenhagen, Copenhagen, Denmark; ^4^Elsass Institute, Charlottenlund, Denmark

## Abstract

Parenting a preterm infant is more challenging than a full-term one. Parent involvement in early intervention programs seems to have positive psychosocial effects on both the child and parent. CareToy is an innovative smart system that provides an intensive individualized home-based family-centred EI in preterm infants between 3 and 9 age-corrected months. A RCT study, preceded by a pilot study, has been recently carried out to evaluate the effects of CareToy intervention on neurodevelopmental outcomes with respect to Standard Care. This study aims at evaluating the effects of CareToy early intervention on parenting stress in preterm infants. Parents (mother and father) of a subgroup of infants enrolled in the RCT filled out a self-report questionnaire on parenting stress (Parenting Stress Index-Short Form (PSI-SF)) before (T0) and after (T1) the CareToy or Standard Care period (4 weeks), according to the allocation of their preterm infant. For twins, an individual questionnaire for each one was filled out. Results obtained from mothers and fathers were separately analysed with nonparametric tests. 44 mothers and 44 fathers of 44 infants (24 CareToy/20 Standard Care) filled out the PSI-SF at T0 and at T1. CareToy intervention was mainly managed by mothers. A significant (*p* < 0.05) reduction in Parental Distress subscale in the CareToy group versus Standard Care was found in the mothers. No differences were found among the fathers. CareToy training seems to be effective in reducing parental distress in mothers, who spent more time on CareToy intervention. These findings confirm the importance of parental involvement in early intervention programs. This trial is registered with Clinical Trial.gov NCT01990183.

## 1. Background

Parental stress can be defined as “a complex process in which adults feel overwhelmed in their role in relation to the responsibilities associated with it” [1]. According to Abidin's parenting stress theory that represents the theoretical framework of this study, the parental stress is “a multidimensional concept which is cumulative, highly influenced by environment, and a result of parent-child transactions that promote negative feelings in parents” [2]. Abidin's parenting stress theory [2, 3] includes three levels of stressors: those arising from the parental domain (e.g., sense of competence as a parent and attachment with a child); the child domain (e.g., adaptability to situations, mood); and the situational, contextual, and social domain (e.g., life events and work environment). These three domains work together to elicit appraisal by parents.

In general, a “physiologic” dose of stress could be motivational, but excessive parental stress may have negative consequences on the child and on the family as a whole [3].

The transition to parenthood is a crucial step whose evolution of both mental and emotional aspects has individual repercussions [4] often more evident in mothers [5] and, as a consequence, alters couple stability [6, 7].

Although stress in its definition is related to the condition of becoming a parent per se, there are some factors which can increase parental stress. Preterm birth is an event with several significant consequences, which can affect the child, parents, and caregiver-infant dyad [8]. A recent meta-analysis [9] has showed that parents and more specifically mainly mothers of preterm infants experience markedly more parental stress compared to parents of term-born infants. This might be related to possible complications of birth, updating the idea that the preterm birth is a source of stress in itself.

Moreover, normal stress of parenthood increases in relation to the frequent problems of a preterm infant, such as medical complications and physical and emotional isolation between parents and infants [10]. Parents with lower gestational age infants probably reported more life stress in relation to long periods spent in neonatology intensive care units (NICUs), which often puts a strain on marital relations [11]. Moreover, due to the needs of the infant, one parent may choose to decrease working hours or to cease working at all, resulting in lower family income [12].

There is evidence pointing to a high correlation between preterm birth and increased parental stress: preterm birth has a negative effect on the relationship between the mother and infant [13–15]; families of preterm infants have frequently reported higher levels of stress [16, 17] than those of full-term infants [9]. Parenting stress in mothers of preterm infants at one year of age is significantly greater than that found in mothers of full-term infants [18].

Most studies have investigated only maternal stress, and there is less evidence about paternal stress. Previous studies in high-risk groups of parents of chronically ill children or children with behavioural problems have shown that mothers and fathers presented similar levels of stress and anxiety [19]. Studies which measure parenting stress in both parents show a correlation between partners' levels of stress [20]. According to this, parental stress has been demonstrated to be mutually shared among family members and acts as a sort of predictor of general family well-being [21].

Moreover, there are several studies showing a relationship between parenting self-efficacy and parenting stress; this suggests that parent outcomes may be a reliable measure of psychoeducational program effectiveness, at least in the short term [22–24].

In relation to the importance of a parental role in infant care, the literature suggests that their involvement in early intervention (EI) programs seems to have positive psychosocial effects both on child and on parents [25].

It is well known that preterm infants need early intervention to improve their outcome [26]. Due to age and following a standard guideline, active involvement and compliance of the family are crucial for maximizing intervention effects. As stated above, there is a relationship between active participation of parents in the intervention of their child and levels of stress. Therefore, it has been hypothesized that a reduction of parental stress, together with parental psychological health, is important for improving efficacy of interventions focused on child behaviour [27]. These findings suggest and justify the assessment of parenting stress as an outcome measure in the evaluation of an early intervention program [28].

It is therefore crucial to detect, identify, and monitor parental stress in order to understand and characterize families so as to prevent negative consequences, especially on child development, and maximize the overall benefits of the intervention.

One of the most widely used scales for the assessment of parental stress is the Parenting Stress Index (PSI) [2, 29–35].

PSI, in its original form (Parenting Stress Index Full Length (PSI-FL)), requires considerable time to be elaborated and, given the long battery of tests to be imposed on infants and parents, could result in missing or incomplete information or misunderstanding of questions by parents leading to unreliable answers. For this reason, in 1995, Abidin developed a short form of PSI (PSI-SF), a 36-item tool based on factor analyses of PSI-FL indicating a three-part solution with three dimensions labelled as Difficult Child (DC), Parental Distress (PD), and Parent-Child Dysfunctional Interaction (P-CDI). The validation of the PSI-SF has been based on two samples of Caucasian primarily married mothers of young children (mean age under 4 years). Correlation between total scores on the long and short forms was quite high (0.87) in these samples. In recent years, many authors have adopted the PSI-SF as an outcome measure [36–38] and it has already been used with mothers of preterm infants [39, 40]. The PSI-SF is nowadays a well validated clinical and research tool which assesses stress associated with parenting.

### 1.1. Present Study

On the basis of the previous theoretical framework and background, the assumption of this study is that a playful early intervention aimed at promoting parent-child interaction using a semistructured tool (CareToy) [41–43] in the home environment could have effects not only on infant development but also on parental stress. A RCT study (Clinical Trial.gov NCT01990183) [44], preceded by a pilot study [45], has been recently carried out and has demonstrated positive effects of CareToy intervention on neurodevelopmental outcomes (i.e., improvement of early motor and visual functions) compared to Standard Care. The aim of this study is to investigate the effects of CareToy early intervention carried out on preterm infants, compared to Standard Care, on parental stress.

## 2. Materials and Methods

### 2.1. Participants

This study is a part of the CareToy project. Through a European consortium, the clinical partners responsible for the assessment of enrolled infants were IRCCS Fondazione Stella Maris in Italy and Elsass Institute in Denmark. This study was approved by the local ethics committees of the two institutions. The inclusion criteria were (i) birth between 28 + 0 and 32 + 6 (weeks + days) of gestational age and (ii) 3 ± 9 age-corrected months who had achieved a predefined cut-off score in gross motor ability derived from the Ages and Stages Questionnaire-Third Edition (ASQ-3, a developmental screening tool to be filled in as a self-reported questionnaire).

The exclusion criteria for the CareToy project were (i) birth weight below the 10th percentile; (ii) brain damage (i.e., intraventricular haemorrhage < grade 1, any degree of periventricular leukomalacia, or brain malformation or severe nonneurological malformations); (iii) any form of seizure; (iv) severe sensory deficits (blindness, deafness); and (v) participation in other experimental rehabilitation studies. Enrolment was carried out in Italy and in Denmark, and parents were selected from families enrolled in the CareToy project.

### 2.2. Intervention

#### 2.2.1. CareToy Intervention

CareToy [41–43] is a modular system based on a traditional baby playpen which has been completely sensorized and defined as a biomechatronic gym with sensorized toys. It is delivered at home and is aimed at providing an intensive, highly customized, home-based, family-centred training program, remotely monitored by a clinical centre. Training is composed of specific goal-directed activities, called CareToy scenarios, remotely planned and periodically upgraded by a rehabilitative staff, according to specific infant needs and progress. Training has a high degree of variability and complexity and is multiaxial, promoting different aspects of motor, cognitive, relational, and visual developments during playtime with parents, who are guided in promoting developmental skills of their infant such as head rotation and gaze movement, manipulation skills, and eye-hand coordination. CareToy scenarios can be variably carried out in the supine, prone, or sitting position.

The system is connected to a clinical centre thanks to a telerehabilitation module with customized software to download activities (CareToy scenarios) and send data to the clinical staff so that they can monitor and upgrade scenarios. Every family has a password corresponding to their personalized program. For the first sessions, the training therapists go to the family's house to teach them how to use the system and, above all, how to interact and play with their child. Afterwards, parents continue on their own with the training, continuously remotely guided for each activity (e.g., figures and diagrams about how to prepare the system and how to position the infant are shown) by the software. In this way, parents play a decisive role in the management of training and are free to choose when and how to play with their infant.

If necessary, therapists are available by phone and, in some cases, to visit homes to provide assistance, but in our experience, this seldom happens and mainly in families with twins.

At the end of each day, the CareToy system automatically sends a training report to the rehabilitation staff, who then can monitor and adjust the system for subsequent training in order to progressively promote more complex abilities when the previous ones have been achieved.

In the aforementioned RCT [44], CareToy training was programmed for a mean daily duration of 30-45 minutes for 4 weeks (a total of 28 days).

#### 2.2.2. Standard Care

As described in detail by Sgandurra et al. [43, 44], Standard Care consisted of a bimonthly follow-up visits, during which current care advice on the early management of preterm infants and booklets dedicated to home care of preterm infants were distributed, according to standard recommendations of Italy and Denmark. Parents were trained on how to manage the care of their infants with illustrated material and counselling by the clinical staff (e.g., about how to handle or stimulate play with their preterm infants). In rare cases, sporadic sessions with a physical therapist for special assistance were arranged. In such cases, the number and type of performed activities were recorded on a dedicated diary.

### 2.3. Outcome Measures

According to study design [38], clinical assessment was performed at baseline (T0, in the week before the 4th week of CareToy Intervention/Standard Care) and in the week after the end of CareToy Intervention/Standard Care period (T1, primary endpoint).

The PSI-SF, a screening instrument for the early identification of parent-child systems which are under stress and at a risk of developing dysfunctional parenting behaviour, is the outcome measure used for addressing the aim of this study.

The PSI-SF consists of 36 items, derived directly from the full-length PSI. Answers are obtained using a Likert scale, which ranged from 1 (Strongly Disagree) to 5 (Strongly Agree). The range of total PSI-SF scores varies from 36 to 180. Scores above the 85th percentile (90 raw score) are considered clinically significant, and higher scores indicate severe levels of parenting stress.

The instrument produces a total stress score indicating the overall level of parenting stress with three subscales: Parental Distress (PD) which measures the level of distress due to the role of mother/father and personal factors; Parent-Child Dysfunctional Interaction (P-CDI) which reflects whether and how the child meets parental expectations; and Difficult Child (DC) which measures the behavioural characteristics of the child that makes them either easy or difficult to manage.

In this study, we presented data using the Parenting Stress Index-Short Form related to the changes detected at primary endpoint (T1) with respect to the baseline (T0). The current data refer to a subgroup of parents (both mothers and fathers) of infants enrolled in the CareToy project who accepted to complete the PSI-SF at T0 and T1.

Mothers and fathers were asked to fill out the questionnaire separately. For twins, an individual questionnaire for each infant was completed.

### 2.4. Statistical Analysis

All statistical analyses were carried out by means of the Statistical Package for Social Sciences (SPSS, version 20.0), with *p* values < 0.05 considered to be statistically significant. Medians and 95% confidence intervals (CI) of infant, mother, and father characteristics and baseline measures for the total sample and separately for each group (CareToy and Standard Care) were calculated to check for baseline differences. Changes in the total stress and in each subscale (PD, P-DCI, and DC) score were calculated separately for the mother and father at baseline (T0) and after the intervention period (T1) for each group. All analyses at baseline and any delta changes (T1-T0) were analysed by means of a Mann–Whitney *U* independent sample test. Chi-square tests were carried out for categorical variables. Finally, a multiple regression analysis for PD delta changes in mothers allocated to the experimental group was used to verify if these changes were related to the combined effect of the PD baseline values at T0 and the hours of CareToy training (predictors). Unstandardized *β* coefficients and significances were reported in addition to model effect sizes (R2) and *p* values.

## 3. Results

### 3.1. Participants

**Figure 1:**
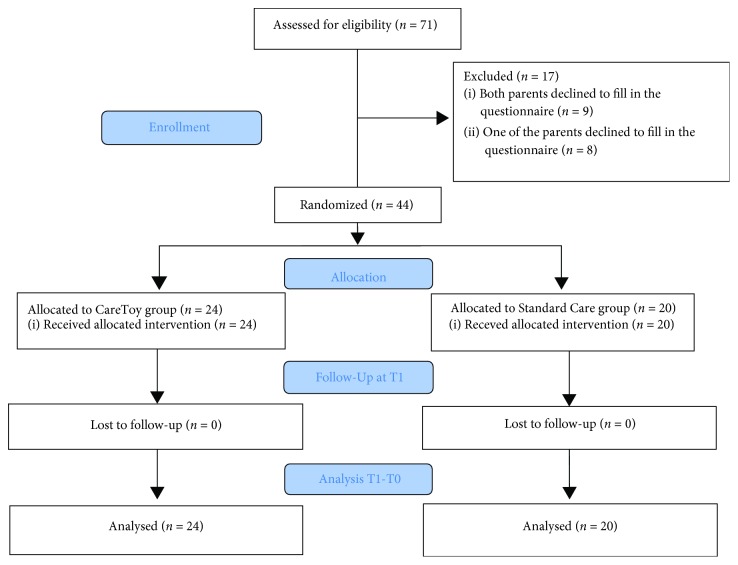


Out of 71 families, a total number of 44 mothers and 44 fathers of 44 infants enrolled in the CareToy project (24 CareToy/20 Standard Care) filled out a PSI-SF at T0 and T1 (Figure 1). The sample was composed of 44 mothers (mean age 37.52 ± 4.48) and 44 fathers (mean age 40.78 ± 5.34). The description of samples of parents and infants is presented in Table 1.

### 3.2. Intervention

#### 3.2.1. CareToy Training

CareToy intervention was performed by all infants considering a minimum drop-out criterion of 51% of planned scenarios in order to include their data in the analysis. The total amount of training had a mean value of 9.55 ± 3.81 hours within the whole group of infants. In the 91.67% (22/24) of cases, CareToy training was mainly performed by mothers.

### 3.3. Outcome Measure

**Figure 2:**
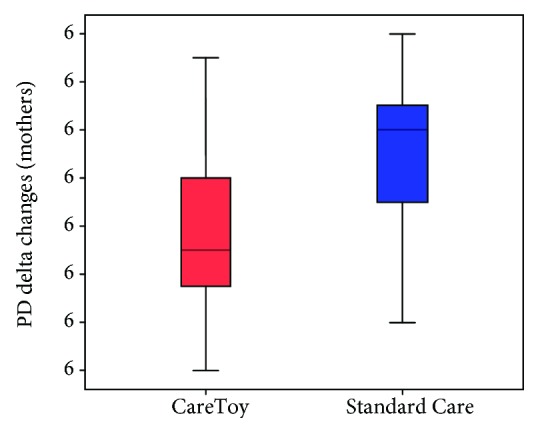


At T0, no differences were found in the total stress and in all subscales scores between mothers and between fathers of the two groups (CareToy vs. Standard Care). No differences were also found between fathers and mothers at T0 considering both total sample and the two groups separately. A significant (*p* < 0.05) reduction in the Parental Distress subscale in the CareToy group versus Standard Care was found in mothers (Figure 2, Tables 2–4) at T1. No difference was found in the other subscales and in the total stress for mothers. Moreover, no difference for all assessed scores was found between fathers of the two groups.

Multiple regression analysis of PD delta changes in mothers allocated to the CareToy group with the two predictors (PD values at baseline and hours of CareToy training) produced *R*^2^ = 0.704, *F*(2, 23) = 27.49, and *p* < 0.001. PD delta change values were significantly correlated with PD at T0 (*β* = −0.504, ES = 0.069, *t* = −7.368, and *p* < 0.001) and for hours of CareToy training (*β* = −0.557, ES = 0.164, *t* = −3.391, and *p* = 0.003).

## 4. Discussion

This paper presents the results of one of the first studies in which parents are the main figures directly involved in infant treatment. In fact, CareToy training, even if planned and remotely monitored by the rehabilitation staff, is actually carried out at home by the parents who are free to choose the best moment, both for them and for their infant, to execute the planned CareToy scenarios.

As expected, thanks mainly to the possibility of obtaining maternal leave from work (as described in a previous work [12]), mothers were more involved and active in training than fathers. Even if the availability of the CareToy system at home potentially allows its use during free time (e.g., evening or weekends), only two fathers took advantage of this to use the system. Moreover, these results are in accordance with the literature, in which paternal stress seems to be lower (Tables 2 and 3) [9] and less affected by the intervention [46, 47].

The different degrees of involvement could explain the different results between mothers and fathers. In fact, at baseline, mothers and fathers had similar scores for the various PSI-SF subscales. After 4 weeks, however, changes in the PD subscale were significant only in the group of mother-infant dyads who carried out CareToy training. Therefore, we can suppose that CareToy intervention plays a role in decreasing the PD subdomain of mothers in the CareToy group.

We might hypothesize that the direct involvement of mothers in carrying out CareToy training enhanced their ability to have stronger positive feelings regarding their role as caregivers. Mothers can perceive that they are promoting the development of their infants under the guidance of a competent driver, i.e., the remote management and control of the rehabilitation staff. The main aspect of CareToy is that the mother, after having completed the instructions of setting up the scenarios, is completely free to play and discover the planned activities with her infant. Furthermore, the mother does not have the burden of thinking how to organize the stimulation activities because the various scenarios automatically propose the goal-directed activities to the mother-infant dyad and automatically change the sequences in relation to the activities performed by the infant.

Another important aspect is that the CareToy telerehabilitation program could be a sort of coaching for the main users, namely, mother-infant dyads. Through the activities experienced during CareToy training, mothers learn how to manage the various positions of her child and how to execute the proposed activities which can then guide her to a better understanding of the goals of the activities. This can, in turn, lead to greater self-confidence, more realistic expectations, and a deeper understanding of her infant's signals and needs.

Moreover, if mothers improve their understanding of the most appropriate stimulation and if infant feedback becomes more positive, a virtuous circle in which stimulation and reward are positively reinforced will be established, as some studies on preterms have already shown [48]. We can hypothesize that, even if CareToy training is directed at infants, involvement of mothers in conducting training represents a sort of behavioural parenting programs. This hypothesis can be supported by the results of this study that are in line with the evidence in meta-analyses on behavioural parenting programs that reduce the parent domain of stress.

**Figure 3:**
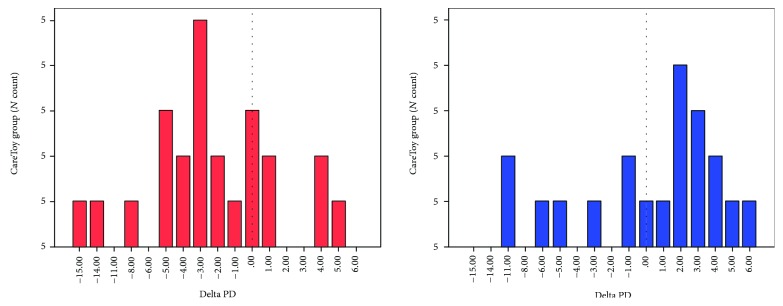


The last important aspect could be related to the direct use of a highly technological device, the CareToy system, instead of a standard baby gym, toys, and traditional rehabilitative tools. Involvement in a scientific and highly resonant research project could increase self-confidence and give the perception of taking upmost care of the infant. This last aspect could also explain the increased parental stress values of the control group because they realized they did not have a chance, at that time, to use the CareToy system with their infants. Examining the single change values (Figure 3), we can see that the range of changes for the CareToy group was significant varying from -15 to +4 and the majority of cases had a decrease of about -5, -4, and -3. For the Standard Care group, we can see that the lack of an early intervention program could cause an increase in the level of stress in some cases.

### 4.1. Limitations and Future Perspectives

In this study, as part of the CareToy project, no inclusion and exclusion criteria were set for parents. In order to improve the quality of future investigations, it would be interesting to adopt some inclusion criteria based on parent cognitive level and record data about the psychopathological history and risk of parents.

## 5. Conclusions

To our knowledge, in the literature, a few articles have evaluated parental stress as an outcome before and after an intervention. Moreover, available data only deal with interventions directly focused on parents or on both infants and parents (e.g., Mother-Infant Transaction Program (MITP); [28]).

This study represents an important starting point for the comprehension of levels of parental stress in relation to their active participation in an intervention focused on their infant. Enabling parents to perform home training may help reduce their feelings of stress on the one hand and maximize infant outcome on the other, and these aspects could have positive consequences on the entire family.

One future perspective could be to investigate parental stress levels also at follow-up points in order to assess medium- and long-term effects of training.

## Figures and Tables

**Table 1:** Description of samples of parents and infants.

Characteristics of the sample	Total	CT group	SC group
Infants	44	24	20
Infant age (months)	3.85 ± 0.93	3.73 ± 0.86	3.99 ± 1.02
Twins	20	12	8
Infant sex (no. of males)	24	15	9
Infant nationality (no. of Italians)	25	13	12
Mother age (years)	37.52 ± 4.48	37.54 ± 4.45	37.50 ± 4.77
Father age (years)	40.78 ± 5.34	40.31 ± 4.97	41.40 ± 6.00
Marital status (no. of married)	30	17	13
Involvement in the training (no. of mothers)		22	

**Table 2:** Baseline (T0) and outcome (T1) values of mothers for the total sample and separately for each group (CT and SC).

PSI-SF domains	Mothers (T0)			Mothers (T1)		
	Total median [95% CI]	CT median [95% CI]	SC median [95% CI]	Total median [95% CI]	CT median [95% CI]	SC median [95% CI]
PD	24.00 [23.02-28.00]	23.00 [22.06-29.86]	25.00 [21.69-28.31]	25.00 [22.36-25.82]	24.50 [20.48-26.02]	26 [22.93-27.17]
P-CDI	22.00 [20.54-23.28]	21.00 [18.96-22.04]	22.00 [21.22-25.83]	20.00 [18.60-21.57]	19.00 [16.98-21.36]	21.00 [19.08-23.21]
DC	21.00 [20.41-23.11]	21.00 [19.16-21.84]	22.00 [20.75-25.63]	21.00 [19.67-22.47]	20.00 [17.72-21.69]	23.00 [20.71-24.53]
Total stress	70.00 [65.21-73.14]	65.00 [61.72-72.19]	73.00 [65.38-78.05]	65.00 [61.72-68.77]	64.00 [57.28-66.97]	67.00 [63.68-73.94]

**Table 3:** Baseline (T0) and outcome (T1) values of fathers for the total sample and separately for each group (CT and SC).

PSI-SF domains	Fathers (T0)			Fathers (T1)		
	Total median [95% CI]	Total median [95% CI]	Total median [95% CI]	Total median [95% CI]	CT median [95% CI]	SC median [95% CI]
PD	22.00 [20.33-24.38]	22.00 [20.33-24.38]	22.00 [20.33-24.38]	21.00 [20.33-23.98]	22.50 [19.58-25.50]	21 [19.48-23.95]
P-CDI	20.00 [19.57-22.07]	20.00 [19.57-22.07]	20.00 [19.57-22.07]	18.00 [18.28-21.10]	19.00 [18.11-22.64]	18.00 [17.17-20.64]
DC	21.00 [20.55-23.49]	21.00 [20.55-23.49]	21.00 [20.55-23.49]	20.00 [19.43-22.43]	19.50 [18.05-23.29]	22.00 [19.76-22.71]
Total stress	63.00 [61.14-69.26]	63.00 [61.14-69.26]	63.00 [61.14-69.26]	62.00 [58.73-66.82]	63.50 [56.71-70.45]	61.00 [57.48-66.23]

**Table 4:** Delta changes (T1-T0) with related *z*-scores and level of significance for mother and father values.

Delta (T1-T0) PSI-SF domains	Mothers			Fathers		
	CT median [95% CI]	SC median [95% CI]	*z* (*p*)^∗^	CT median [95% CI]	SC median [95% CI]	*z* (*p*)^∗^
PD	-3 [-4.73/-0.68]	2 [2.12/2.22]	-2.237 (0.025)	1 [-1.42/1.59]	1 [2.55/1.50]	-0.126 (0.900)
P-CDI	-0.50 [-3.47/0.80]	-2 [-4.06/-0.71]	-0.925 (0.355)	-1 [-2.82/1.15]	-1 [-2.93/0.02]	-0.435 (0.663)
DC	-1 [-2.06/0.48]	0.00 [-2.10/0.96]	-0.046 (0.963)	-0.50 [-2.51/0.18]	-1 [-2.31/0.31]	-0.011 (0.991)
Total stress	-3.50 [-8.26/-1.41]	-2.00 [-6.44/0.63]	-0.592 (0.554)	1 [-5.53/1.69]	1 [-6.52/0.52]	-0.514 (0.607)

^∗^Mann-Whitney nonparametric test.

## Data Availability

All the data used to support the findings of this study are included in the article.
